# Narrative Reminder Recall to Improve Pediatric Influenza Vaccination: A Pilot Randomized Clinical Trial

**DOI:** 10.1001/jamanetworkopen.2025.52149

**Published:** 2026-01-06

**Authors:** Joshua T. B. Williams, Kate Kurlandsky, Amy B. Stein, Neha Bharadwaj, Simon J. Hambidge, Rocio I. Pereira, Sonja C. O’Leary, Deidre Johnson, Sean T. O’Leary

**Affiliations:** 1 Denver Health, Denver, Colorado; 2 Department of Pediatrics, University of Colorado School of Medicine, Aurora; 3 Center for Health Systems Research, Denver Health, Denver, Colorado; 4 Department of Medicine, Division of Hospital Medicine, University of Colorado School of Medicine, Aurora; 5 Department of Medicine, Division of Endocrinology, Metabolism & Diabetes, University of Colorado School of Medicine, Aurora; 6 Center for African American Health, Denver, Colorado; 7 Adult & Child Center for Outcomes Research and Delivery Science, Aurora, Colorado

## Abstract

**Importance:**

Seasonal influenza causes morbidity and mortality in vulnerable communities. Narrative reminder recall (ie, digital stories using multimodal narratives that uplift community voices) may be scalable as an equity-promoting intervention to increase vaccination rates.

**Objectives:**

To assess (1) the reach of texts with pediatric influenza vaccine digital stories among caregivers of young children and (2) associations of caregiver intervention assignment with time to child influenza vaccination.

**Design, Setting, and Participants:**

This pilot randomized clinical trial included caregivers and their children (aged 6 months to 5 years) from 2 safety-net clinics in historically Black neighborhoods in Denver, Colorado between September 16, 2024, and March 31, 2025. Caregivers aged 18 years and older who preferred English and their children, provided they had 1 well visit in the past 18 months and would be aged 6 to 71 months during the influenza season, were included.

**Intervention:**

The digital storytelling intervention included texts with links to 5 stories (3 featuring caregivers who identified as Black or African American) sent in October 2024. Usual care included 1 message sent via an electronic health record messaging portal to caregivers of all children eligible for influenza vaccines.

**Main Outcomes and Measures:**

The primary outcome was digital story reach (ie, the proportion of caregivers viewing 1 or more digital stories for any length of time). Time to first influenza vaccination was recorded for children. Adjusted Cox proportional hazards modeling (with 95% CI) fit time to vaccination by group and baseline covariates. Kaplan-Meier curves analyzed the proportions of children remaining unvaccinated by group over time.

**Results:**

Intention-to-treat analyses included 200 children (100 per group; mean [SD] age, 29.1 [16.1] months; 96 male [48%]; 77 Black or African American [39%]; 90 Hispanic or Latino/a [45%]; 54 White [27%]) and their 198 caregivers (100 usual care; 98 intervention; mean [SD] age, 30.5 [7.5] years; 176 mothers [89%]; 77 Black or African American [39%]; 87 Hispanic or Latino/a [44%]; 25 White [13%]). Overall, 686 of 686 texts (100%) were delivered; digital stories were viewed by 7 caregivers (7%). More children of intervention caregivers were vaccinated when influenza activity peaked on February 15, 2025, (percentage unvaccinated, 62% [95% CI, 53%-72%] in intervention vs 74% [95% CI, 66%-83%] in usual care; Z test, 1.82; *P* = .03). In adjusted analyses, children of intervention caregivers were 63% more likely to be vaccinated compared with children of caregivers receiving usual care (adjusted hazard ratio, 1.63; 95% CI, 1.01-2.64).

**Conclusions and Relevance:**

In this pilot randomized clinical trial of a text message digital storytelling intervention centering Black families, intervention receipt was associated with improved pediatric influenza vaccination, although intervention reach was low.

**Trial Registration:**

ClinicalTrials.gov Identifier: NCT06274359

## Key Points

**Question:**

Is a text message and digital storytelling intervention for caregivers to address influenza vaccination in children feasible?

**Findings:**

In this pilot randomized clinical trial with 200 children and 198 caregivers in a safety-net health system during the 2024-2025 influenza season, 100% of texts reached intervention caregivers, and 7% of caregivers watched 1 or more digital stories. Children of intervention recipients were 63% more likely to be vaccinated compared with children of caregivers receiving usual care.

**Meaning:**

In this pilot randomized clinical trial, a cocreated, text message storytelling intervention was associated with improved pediatric influenza vaccination coverage, but digital stories had limited reach.

## Teaser

This pilot randomized clinical trial examines the reach of a text message–based digital story intervention for caregivers to encourage pediatric influenza vaccination.

## Introduction

Every winter, millions of US children are infected with influenza, tens of thousands are hospitalized, and hundreds or more die.^1^ Children younger than 5 years and those with chronic medical conditions are at highest risk of severe illness and death. Studies of pandemic and seasonal influenza have also found children from racial and ethnic minority groups are 2 to 3 times more likely to get sick, be hospitalized, and die from influenza.^2,3,4,5,6^ Disparities in morbidity and mortality are associated with disparities in influenza prevention. The seasonal influenza vaccine is recommended for all children aged 6 months old and older,^7^ but studies using diverse methods over the last decade have found large disparities in vaccination coverage, including for Black children.^8,9,10,11^ Concerningly, influenza vaccination coverage for all young children has declined dramatically since the COVID-19 pandemic.^12^

Multilevel risk factors contribute to vaccination inequities^13,14,15,16,17^; vaccine hesitancy and mistrust are key concerns.^18,19^ Experts have called for research to develop culturally competent messages that center affected families and speak to historical mistrust.^20^ Digital stories are multimodal narratives grounded in the voices and experiences of community members,^21^ and they have been effective in clinical trials to treat hypertension and diabetes in Black and Latino adults.^22,23^ Recently, they have been piloted to improve confidence in human papillomavirus vaccines.^24,25,26^ Previously, we conducted qualitative interviews with caregivers and staff to explore whether digital storytelling could be an intervention strategy to improve vaccine confidence in a federally qualified health center with large influenza vaccination disparities for Black children.^27^ Caregivers found digital stories relatable, and staff felt they could be feasibly implemented via text messages.^27^ Subsequent work in partnership with a Black health nonprofit organization lead to the cocreation of digital stories that centered Black families’ experiences with influenza.^28^

The aims of this pragmatic (ie, concerned with producing answers to questions faced by immunization delivery decision-makers about best practices for improving influenza vaccination equity) pilot clinical trial were to (1) assess the reach of text messages and pediatric influenza digital stories among caregivers of young children in 2 urban clinics and (2) examine associations of caregiver assignment to the text message digital storytelling intervention with their child’s time to influenza vaccination in the 2024-2025 season, compared with dyads assigned to receive usual care.

## Methods

### Design, Setting, and Participant Recruitment

This was a pilot randomized clinical trial of text messages with links to pediatric influenza digital stories among caregivers and their children aged 6 months to 5 years, with reporting following the Consolidated Standards of Reporting Trials (CONSORT) reporting guideline for randomized clinical trials.^29^ The study was conducted at 2 federally qualified health centers in historically Black neighborhoods in Denver, Colorado.^30^ Both clinics have disparities in seasonal influenza vaccination coverage and have invested partners who desired to creatively work to improve vaccination equity.^27^ In the 2023-2024 season, the clinics cared for 2662 Black children aged 0 to 19 years and administered 672 influenza vaccines (coverage: 25%). The study protocol (Supplement 1) was approved by the Colorado Multiple institutional review board and registered on ClinicalTrials.gov (NCT06274359). The overarching study goal was to inform the feasibility of a future multisite trial of digital storytelling (sent via text messages) to improve influenza vaccination equity.

Caregivers were approached for participation if their child was empaneled (ie, had 1 well child visit in last 18 months) at the time of recruitment, would be aged 6 months to 5 years at any time during the upcoming influenza season (defined a priori as September 16, 2024, to March 31, 2025), and had a scheduled visit at either clinic during the recruitment period. Caregivers whose first language was not English (digital stories were narrated in English), were younger than 18 years, or who had children with medical contraindications to influenza vaccination were excluded.

A professional research assistant or the principal investigator contacted caregivers of children with scheduled appointments at study clinics between July 1 and September 15, 2024, on the day prior to their scheduled appointment by phone, inviting them to participate. If the caregiver did not pick up, a message was left with a callback number and email address. A maximum of 3 attempts were made. Caregivers provided verbal consent to participate in the study prior to completing a baseline survey via REDCap^31^ with attitudinal questions about influenza and influenza vaccines and sociodemographic information, including race and ethnicity (American Indian or Alaska Native, Asian, Black or African American, Hispanic or Latino/a, Middle Eastern or North African, White, and other [a discrete race and ethnicity category from which to choose]), which was self-reported by caregivers for themselves and their children and recorded in the baseline survey. Information for caregivers who refused participation was extracted from the child’s electronic medical record if available and preserved in aggregated fashion. A $20 incentive was mailed to participants.

### Intervention Development and Implementation Plan

The intervention was developed via rapid community translation as described previously.^28^ Briefly, the intervention involved a series of 7 brief text messages delivered via REDCap Twilio software^31^ to caregivers’ preferred cell phones. The first message included community messaging with a digital story centering a Black caregiver and her son, followed immediately by a second text message with vaccination appointment scheduling information. Subsequent messages followed a sequence crafted by community members using cocreated language and contained community members’ digital narratives about influenza and influenza vaccines.^28^ Of the 5 digital stories, 3 centered Black mothers and 2 included storytellers of other races, genders, and roles; most stories emphasized the severity of influenza disease and lasted 2 to 3 minutes.^28^ For example, in the first digital story sent out, a mother described her son’s multiple hospitalizations and remembered how the influenza vaccine seemed like an ace up his sleeve—or a strong hand—that kept him in school and on track to graduate and pursue his life’s goals.^28^ Accompanying text read: “Prevent what you can. See how [name] protected her son with a flu vaccine—an Ace up his sleeve—each winter. [Video link]”^28^ All 5 narratives with associated community-cocreated messaging have been described previously.^28^

Text messages were sent weekly on Wednesdays between 10:00 AM and 2:00 PM, with a reminder text message with scheduling information sent following the third digital story. Stories were hosted privately on Vimeo, with each caregiver receiving a unique link to every story, facilitating the assessment of video reach at the individual caregiver level. Texts were delivered from October 2 to October 25, 2024.

### Sample Size Calculation and Randomization

We did not anticipate full power to detect a significant treatment effect in this pilot study. Thus, we evaluated potential sample sizes based on the precision for estimating the intervention’s effect for a future multisite trial, measured by the difference in the proportion of children receiving 1 or more influenza vaccines between intervention and control groups. With a sample size of 200 children and an intervention effect size of 0.10, we calculated we could attain a margin of error (equal to one-half of the width of a 95% CI) of 0.14 on the proportion of children vaccinated. Because recruiting additional children minimally reduced the margin of error (approximately 0.01 per additional 50 children), our recruitment goal was 200 children. Calculations assumed equal allocation between groups and a proportion vaccinating among controls of 0.50.

After recruitment was completed, caregivers were block randomized on 3 covariates: race and ethnicity (Black vs another race or ethnicity), influenza vaccination hesitancy status (hesitant vs not hesitant), and child’s age (<24 months at recruitment vs 24-71 months). Randomization occurred—with the biostatistician blinded to treatment group—from September 16 to October 1, 2024, with 100 children assigned to each group: intervention vs usual care. Usual care included 1 message sent the week of September 23, 2024, via an electronic health record messaging portal to caregivers of all children eligible for influenza vaccines, noting that COVID-19 and influenza vaccines were available; parents needed to log in to view the message.

### Main Outcomes and Measures

The primary caregiver outcome was digital story reach, defined as the proportion of caregivers viewing 1 or more digital stories for any length of time. Text message reach among intervention caregivers was also assessed via Twilio functionality, which tracks whether a message was sent to an account holder’s phone successfully (but does not provide information on whether the account holder actually read the text). Caregiver independent variables included age in years, sex (as a biological variable), educational attainment, race and ethnicity, relationship to child, number of living children, and mean influenza Vaccine Hesitancy Score (VHS), as validated by Helmkamp et al^32^ (mean ≥3 indicates hesitancy).

The primary child outcomes were receipt of 1 or more influenza vaccinations during the influenza season and time to first vaccination. At the time of the study, official guidance recommended 2 vaccine doses for children younger than 9 years who had not yet received 2 doses prior to the season.^7^ We defined vaccination in a binary fashion because prior studies in our health system found caregiver vaccine hesitancy increased a child’s risk of influenza nonvaccination but did not increase their risk of partial vaccination (vs full vaccination).^19^ We chose time to first vaccination because influenza vaccination is always relative to seasonal influenza activity; time to first influenza vaccination was assessed beginning on September 16, 2024, the date influenza vaccines were first available in our health system and a best practice advisory for child influenza vaccination was launched. Child independent variables were age (months), sex (biological variable), race and ethnicity, number of visits at the medical home during influenza season, and vaccination dates. Vaccine-only visits were not included as a medical home visit because only 1 child had this visit type during the study period (and the child had 3 separate visits with clinicians at the medical home during the study period).

### Statistical Analysis

To assess recruitment bias, we compared participating caregivers with those who refused participation on independent covariates of interest. Next, we calculated descriptive analyses for participating caregivers and children on independent variables of interest, stratified by study group, using *t* test of means or categorical comparisons. Attitudinal data on influenza disease and the influenza vaccine were analyzed and presented categorically, with responses stratified by caregiver VHS status (hesitant or not hesitant). Likert scale item comparisons were conducted via Mann-Whitney *U* testing. The crude proportions of caregivers whose REDCap data indicated successful text message receipt and who watched 1 or more digital stories and were calculated.

Next, the crude proportion of children who received 1 or more influenza vaccines during the influenza season were calculated, with disparities calculated between racial and ethnic groups. The proportions of children remaining unvaccinated over time, stratified by group, were graphed via Kaplan-Meier survival curves and compared (a log rank *P* < .05 was significant). One time point was selected a priori for treatment group comparison: peak influenza activity, defined as 21.7 weeks from study start, or February 15, 2025, per local data.^33^ The proportions remaining unvaccinated between groups were compared with a 1-sided, 2-sample z test.

Unadjusted and adjusted Cox proportional hazards regression analyses with 95% CIs assessed the likelihood of vaccination at any time in the influenza season by intervention group and 5 predefined covariates that were deemed clinically relevant: caregiver influenza VHS, caregiver race and ethnicity, caregiver education, child medical home visits during the season, and child age group. Caregiver race and ethnicity was included because racial concordance with storytellers was perceived as an important potential determinant of digital story engagement in qualitative interviews.^27^ Intention to treat analyses included all 200 children and 198 caregivers. Model assumptions were evaluated during analyses, and covariates were assessed for collinearity based on pairwise correlations and by variance inflation factors; model assumptions were met and there was no evidence that collinearity affected estimates. Analyses occurred in R Version 4.0 (R Project for Statistical Computing).

## Results

**Figure 1.**
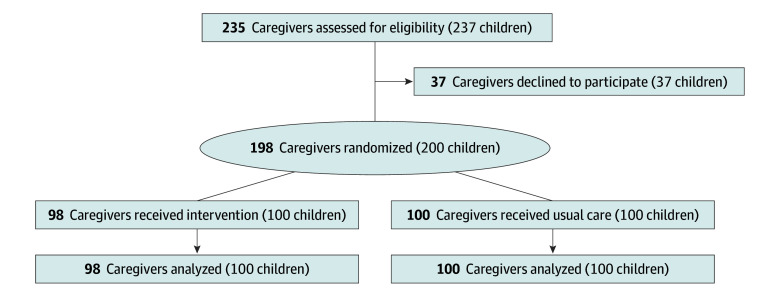
Flow Diagram of Participating Children and Caregivers

Overall, 235 caregivers were approached for participation (Figure 1), and 198 caregivers (100 usual care; 98 intervention; mean [SD] age, 30.5 [7.5] years; 176 mothers [89%]; 77 Black or African American [39%]; 87 Hispanic or Latino/a [44%]; 25 White [13%]) and their 200 children (100 per group; mean [SD] age, 29.1 [16.1] months; 96 male [48%]; 77 Black or African American [39%]; 90 Hispanic or Latino/a [45%]; 54 White [27%]) were enrolled. The eTable in Supplement 2 compares participating caregivers with those who refused participation on covariates of age group, role, and race and ethnicity. Differences in age distribution and role were observed, likely due to missing data (eTable in Supplement 2). The proportions of caregivers who identified as Black among participants and those who refused participation were similar (77 of 198 individuals [39%] vs 12 of 37 individuals [32%]; *P* = .58).

Demographic information for children and caregivers, stratified by treatment group, is in Table 1. Baseline characteristics were similar, including the proportion of caregivers whose VHS mean scores indicated hesitancy about influenza vaccines (21 caregivers [21%] in intervention vs 21 caregivers [21%] in usual care) and the proportions of children without a medical home visit in season (39 children [39%] in intervention vs 45 children [45%] in usual care).

**Table 1.** Sociodemographic Information for Study Participants^a^

Variable and level	All participants, No. (%) (N = 198 caregivers; 200 children)	Study group, No. (%)	
		Intervention (n = 98 caregivers; 100 children)	Usual care (n = 100 caregivers; 100 children)
Caregiver characteristics			
Age, mean (SD), y	30.5 (7.5)	30.8 (7.6)	30.2 (7.4)
Role			
Mother	176 (89)	86 (88)	90 (90)
Father	16 (8)	8 (8)	8 (8)
Other	6 (3)	4 (4)	2 (2)
Education			
≤High school or GED	104 (53)	52 (53)	52 (52)
Some college or bachelor’s	86 (43)	43 (44)	43 (43)
Advanced degree	8 (4)	3 (3)	5 (5)
Race and ethnicity^b^			
American Indian or Alaska Native	7 (4)	3 (3)	4 (4)
Asian	8 (4)	5 (5)	3 (3)
Black or African American	77 (39)	34 (35)	43 (43)
Hispanic or Latino/a	87 (44)	44 (45)	43 (43)
Middle Eastern or North African	1 (1)	0	1 (1)
Native Hawaiian or Pacific Islander	5 (3)	2 (2)	3 (3)
White	25 (13)	13 (13)	12 (12)
Other^c^	6 (3)	4 (4)	2 (2)
Missing or prefer not to answer	2 (1)	2 (2)	0
Vaccine hesitancy score (influenza)			
Not hesitant (mean ≤3)	156 (79)	77 (79)	79 (79)
Hesitant (mean >3)	42 (21)	21 (21)	21 (21)
Child characteristics			
Sex			
Male	96 (48)	53 (53)	43 (43)
Female	104 (52)	47 (47)	57 (57)
Age, mean (SD), mo	29.1 (16.1)	28.1 (15.4)	30.0 (16.7)
Race and ethnicity^b^			
American Indian or Alaska Native	4 (2)	2 (2)	2 (2)
Asian	5 (3)	5 (5)	0
Black or African American	77 (39)	38 (38)	39 (39)
Hispanic or Latino/a	90 (45)	47 (47)	43 (43)
Middle Eastern or North African	0	0	0
Native Hawaiian or Pacific Islander	5 (3)	2 (2)	3 (3)
White	54 (27)	30 (30)	24 (24)
Other^c^	26 (13)	12 (12)	14 (14)
Missing or prefer not to answer	9 (5)	4 (4)	5 (5)
Firstborn child	72 (36)	39 (39)	33 (33)
No. of medical home visits in season			
0	84 (42)	39 (39)	45 (45)
≥1 (vaccine-only visits not included)	116 (58)	61 (61)	55 (55)

Abbreviation: GED, general educational development.

^a^ In 2 cases, children were recruited as siblings because both were in the eligible age range; siblings in the same family unit were assigned to the same treatment group: intervention.

^b^ Categories include race and ethnicity categories marked alone or in combination. Sums exceed 100% because caregivers were allowed to select any and all race and ethnicity categories that applied.

^c^ Other was a discrete race and ethnicity response category from which to choose.

**Figure 2.**
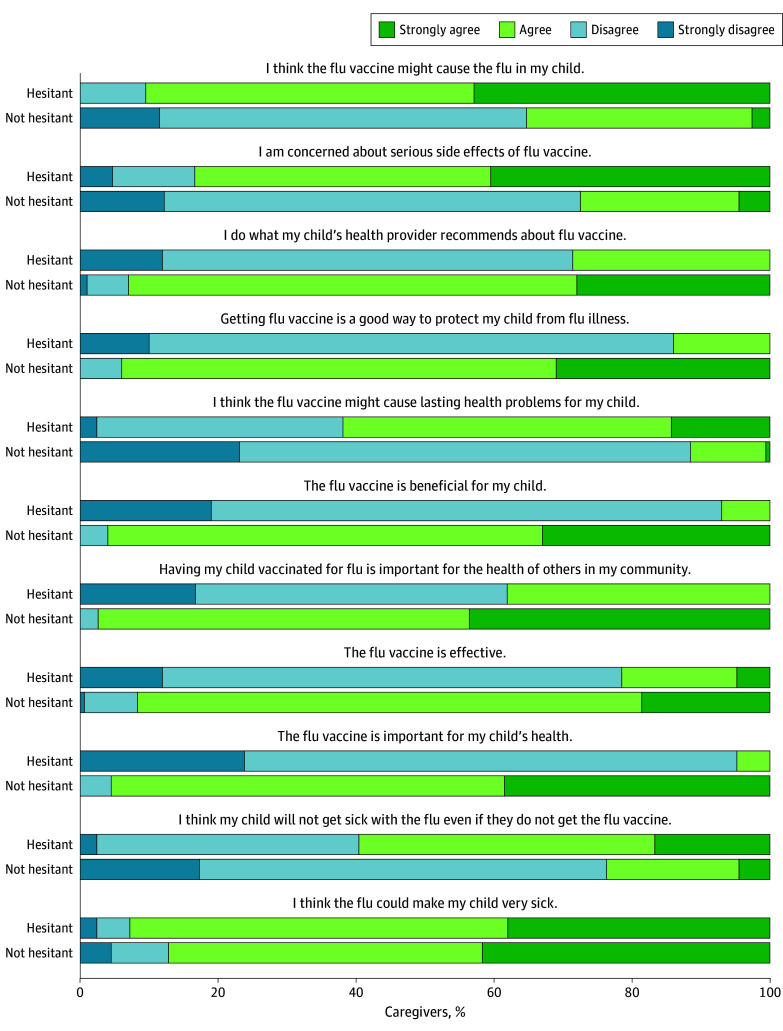
Caregivers’ Responses to Attitudinal Survey Questions About Influenza and Influenza Vaccines, Stratified by Caregiver Hesitancy All comparisons of Likert scale responses via Mann-Whitney *U* testing were statistically different (*W* [range]: 208-1891), with the exception of the last item (*W* = 3283; *P* = .98).

Caregivers’ responses to the VHS influenza questions are presented in Figure 2, stratified by hesitancy. Perceptions of influenza severity were similar between groups, with 39 of 42 hesitant caregivers (93%) and 137 of 156 nonhesitant caregivers (88%) strongly agreeing or agreeing that “the flu could make my child very sick” (*W* = 3283; *P* = .98). Perceptions of influenza vaccines differed by caregiver hesitancy status (range of *W*: 208-1891), although 16 of 42 hesitant caregivers (38%) agreed that “having my child vaccinated for flu is important for the health of others in my community” and 12 of 42 caregivers (29%) agreed they still “do what my child’s health care provider recommends about the flu vaccine.”

Twilio functionality in REDCap indicated 686 of 686 texts (100%) were successfully delivered to all 98 intervention caregivers in October 2024. Vimeo analytic data indicated digital stories reached 7 of 98 intervention caregivers (7%); of these, 1 caregiver viewed all 5 digital stories, 1 caregiver viewed 2 digital stories, and 5 caregivers viewed the first digital story only. Stories were watched for a total of 916 seconds, with a median (range) watch time for any individual story of 52 (2-230) seconds. All 7 caregivers who viewed stories were not hesitant about the influenza vaccine.

Overall, 41 children of intervention caregivers (41%) and 28 children of usual care caregivers (28%) received 1 or more vaccines by season’s end. Overall, 12 of 38 Black or African American children of intervention recipients (32%) received 1 or more vaccines, while 17 of 39 Black or African American children of caregivers assigned to usual care (18%) received 1 or more influenza vaccines. Statistical comparisons among subgroups identifying with 1 race or ethnicity were not performed because the study was not powered to detect differences for subgroups with small sample sizes.

**Figure 3.**
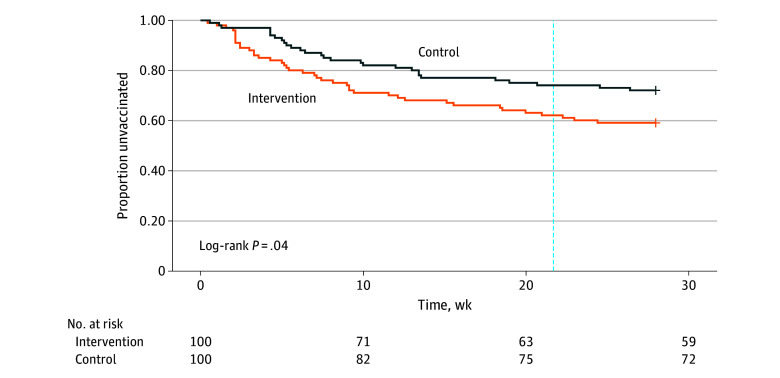
Time to Vaccination, Stratified by Caregiver Study Group Peak influenza activity (ie, 21.7 weeks from the start of influenza season) is represented by the vertical dashed line.

The proportions of children remaining unvaccinated diverged by study group shortly after the intervention began (Figure 3). At 21.7 weeks, or peak influenza activity in Colorado, the proportions of unvaccinated children differed by group (62% [95% CI, 53%-72%] in intervention vs 74% [95% CI, 66%-83%] in usual care; *Z* test value, 1.82; *P* = .03). In unadjusted and adjusted Cox proportional hazards modeling, compared with children of caregivers assigned to usual care, children of intervention recipients were 63% more likely to be vaccinated during the influenza season (adjusted hazard ratio, 1.63; 95% CI, 1.01-2.64). Children with 1 or more visits at their medical home were more likely to be vaccinated than children without any visits (adjusted hazard ratio, 5.13; 95% CI, 2.60-10.13). Additional unadjusted and adjusted hazards of vaccination by covariates of interest are presented in Table 2.

**Table 2.** Likelihood of First Influenza Vaccination Dose at Any Time Point in the Influenza Season^a^

Independent variable and level	Likelihood of first influenza vaccination, HR (95% CI)	Likelihood of first influenza vaccination, aHR (95% CI)
Caregiver study group		
Usual care	1 [Reference]	1 [Reference]
Intervention	1.64 (1.01-2.65)	1.63 (1.01-2.64)
Caregiver VHS status		
Not hesitant (VHS≤3)	1 [Reference]	1 [Reference]
Hesitant (VHS>3)	0.31 (0.13-0.71)	0.30 (0.13-0.69)
Caregiver race and ethnicity		
Black or African American	1 [Reference]	1 [Reference]
Another race or ethnicity^b^	1.41 (0.85-2.34)	1.11 (0.67-1.86)
Caregiver educational attainment		
≤High school or GED	1 [Reference]	1 [Reference]
Some college or bachelor’s	1.19 (0.73-1.96)	1.26 (0.77-2.07)
Advanced degree	4.22 (1.76-10.15)	4.32 (1.75-10.64)
No. of child medical home visits		
0	1 [Reference]	1 [Reference]
≥1	5.50 (2.81-10.75)	5.13 (2.60-10.13)
Child’s age at recruitment, mo		
<24	1 [Reference]	1 [Reference]
24-71	0.37 (0.23-0.60)	0.49 (0.30-0.80)

Abbreviations: aHR, adjusted hazard ratio; GED, general educational development; HR, hazard ratio; VHS, vaccine hesitancy score.

^a^ The influenza season was defined as September 16, 2024, to March 31, 2025. Independent variables were predefined. Analyses were adjusted for caregiver study group, caregiver influenza vaccine hesitancy score status, caregiver race and ethnicity, caregiver educational attainment, child number of visits at their medical home, and child’s age at recruitment.

^b^ Included American Indian or Alaska Native, Asian, Hispanic or Latino/a, Middle Eastern or North African, White, and other (a discrete race and ethnicity category from which to choose).

## Discussion

In this randomized pragmatic pilot trial of caregiver-child dyads recruited from 2 safety-net clinics in historically Black neighborhoods in Denver, Colorado, children of caregivers who received cocreated texts and stories had an increased hazard of vaccination during the influenza season and a decreased proportion remained unvaccinated at peak influenza activity. However, few caregivers engaged with digital stories, and those who engaged with stories did so for various lengths of time, suggesting digital storytelling may require additional adaptations prior to widespread implementation.

First, findings suggest community cocreated text message interventions may still influence caregiver vaccination behaviors, with a modest effect size. For 2 decades, text message reminders to caregivers have been an effective implementation strategy to improve pediatric influenza vaccination rates.^34,35^ However, 2 recent trials of text messages to improve influenza vaccination—one prior to and one shortly after the COVID-19 pandemic—had no effect^36^ or a minimal one ( approximately 1%)^37^ in a subgroup of unvaccinated patients with upcoming appointments only. Because this trial’s comparator group did not contain alternative text messaging content, we cannot definitively attribute intervention effectiveness to text message content; however, we hypothesize poor effectiveness in prior studies may relate to message content.

For example, the first trial during the 2017-2018 influenza season sent messages from the New York State Health Department emphasizing that children “need the flu shot,” “the flu vaccine is safe,” and that “the flu virus changes every year.”^36^ The second trial based messages on a study of older White females’ preferences for influenza vaccination nudges, noting an influenza shot was “reserved for [child’s name]” and instructed parents to “claim [child’s name]’s dose today.”^37^ Conversely, messages in this trial were community cocreated and centered experiences from Black families and children who had influenza. While messages and stories indirectly emphasized influenza severity (which aligned well with attitudinal data regarding influenza among hesitant and nonhesitant caregivers), generic statements about influenza severity were purposefully avoided based on caregiver feedback in preceding interviews.^27^ We did not power this pilot study to compare vaccination rates by racial and ethnic groups; appropriately powered future studies can determine whether the halving of inequities between Black and White children we observed in the intervention group were due to chance. We recommend study leadership involve those who experience disparities as critical partners in the development of future interventions intended to address them.^38^

Despite embedded links to pediatric influenza digital stories in texts, few caregivers viewed the stories, with variable lengths of engagement for those who did. Multiple reasons for the limited reach of stories are possible. First, the need to click on an embedded hyperlink to visualize digital stories may have been a barrier to story engagement (vs embedding the story itself in the message). Second, messages may have been perceived as spam and ignored, especially by vaccine hesitant caregivers (none of whom engaged with a story). Despite these barriers, an engagement rate of 7% is similar to the 0.5% to 6.0% rates observed for followers of social media posts.^39,40^ This suggests, as an implementation strategy,^41^ texts from our health system may still yield comparable engagement as popular social media platforms.

Finally, while the intervention was associated with increased vaccination, overall, children with 1 or more visits at their medical home were more likely to be vaccinated during the season than those who were not seen. Because the proportions of children in intervention and usual care groups with 1 or more medical home visits during the season were comparable, this finding is especially relevant. Access to a medical home has long been associated with immunization timeliness, including within the health system in which this trial was conducted. In 2004, a cluster randomized trial of 2843 infants found that a well child visit intervention significantly increased up-to-date vaccination rates by age 12 months.^42^ Multilevel, sustainable efforts to recall children in midwinter for influenza vaccines are needed and could be an opportunity for anticipatory guidance that is difficult in busy well visits.^43^ Such visits may benefit preschoolers, who had a decreased likelihood of vaccination in this study (compared with infants or toddlers) and only visit clinicians for well checks yearly, possibly missing the influenza season completely.

### Limitations

Limitations include the single site nature of this clinical trial, the exclusion of families who preferred Spanish or another language, and the lack of detailed perceptions from intervention caregivers about the impact of digital stories on their vaccination decisions. Additionally, the use of electronic portal messaging in the usual care group represents a strong difference in implementation of the intervention vs control. Furthermore, the low vaccine coverage in both groups in this study is concerning for translation of the intervention outside of a pilot-controlled trial because caregivers who consent to research may differ from those who decline participation.

## Conclusions

In this pilot randomized clinical trial, cocreated texts and digital stories, as well as medical home visitation, were associated with timely pediatric influenza vaccination. However, the intervention had limited reach. Work to creatively recall families for midwinter visits and refine text-based digital storytelling campaigns for implementation in diverse health systems are warranted.

## Notes

Supplement 1. Trial Protocol

Supplement 2. Data Sharing Statement
