# Supplementary material for: Narrative Reminder Recall to Improve Pediatric Influenza Vaccination: A Pilot Randomized Clinical Trial
Source: JAMA Netw Open. 2026 Jan 6;9(1):e2552149. doi: 10.1001/jamanetworkopen.2025.52149 (PMC12776203; doi:10.1001/jamanetworkopen.2025.52149)
Supplement: Supplement 1. — Trial Protocol [file jamanetwopen-e2552149-s001.pdf]

Protocol #: 21-3985
Project Title: Digital Storytelling to Reduce Pediatric Influenza Vaccination Disparities: A Pilot Pragmatic Trial
Principal Investigator: Joshua T.B. Williams, MD
Version Date: 1/4/2024

I. Hypotheses and Specific Aims:

Digital stories are personal narratives that synthesize images, video, voice, or text to create compelling accounts of human experience. In pilot pragmatic trials, digital stories have improved hypertension control in African American adults and increased self-report of Human Papillomavirus vaccination in Korean women. The 3 specific aims of this proposal are to (1) explore diverse stakeholders' perceptions of and priorities for a digital storytelling intervention through qualitative interviews, (2) co-create a digital storytelling pediatric influenza vaccination intervention through novel community-engaged methods, and (3) assess the feasibility, acceptability, appropriateness, and pilot effectiveness of the intervention in a pragmatic trial in 2 safety-net clinics located in historically Black neighborhoods in Denver, CO.

II. Background and Significance:

The COVID-19 pandemic has highlighted health disparities, but Black children have unduly suffered and died from seasonal influenza for decades.[20] Most years, ~10 million children get influenza, ~50,000 are hospitalized, and 300-500 die.[22] All children age 5 years and younger are at highest risk of severe illness and death, but many studies of pandemic and seasonal influenza have found that Black children are 2-3 times more likely to get sick, be hospitalized, and die than children of other races and ethnicities.[23–27] *These disparities in morbidity and mortality are associated with disparities in influenza prevention.* The seasonal influenza vaccine is recommended for all children aged ≥6 months,[28] but nationwide studies using diverse methods over the last decade have found Black children consistently lag behind children of other races and ethnicities in vaccination coverage.[29–34] **Reducing vaccination disparities is key to reducing influenza disparities in Black children.**

Multi-level risk factors contribute to pediatric vaccination inequities. Poverty, large family size, access to care, distrust of healthcare, health illiteracy, vaccine shortages, and vaccine hesitancy have all been associated with vaccination inequities.[18,39,51–55] In my own work, 42% of unvaccinated inpatients with influenza at a tertiary children's hospital from 2010-14 had ≥1 missed vaccination opportunity.[3] In my survey of 255 parents of 2 year-olds at 3 safety-net clinics in 2019, 25% of Black caregivers were vaccine-hesitant;[6] children of hesitant parents had a 3-fold risk of being unvaccinated for influenza by season's end (Williams et al., unpublished). **Figure 2, an adapted multi-level disparities**

framework,[48,56] shows how risk factors like structural racism[57,58] or vaccine hesitancy contribute to vaccination disparities, with possible interventions at each risk level.

Figure 2. Adapted multi-level disparities framework levels, risk factors & interventions.

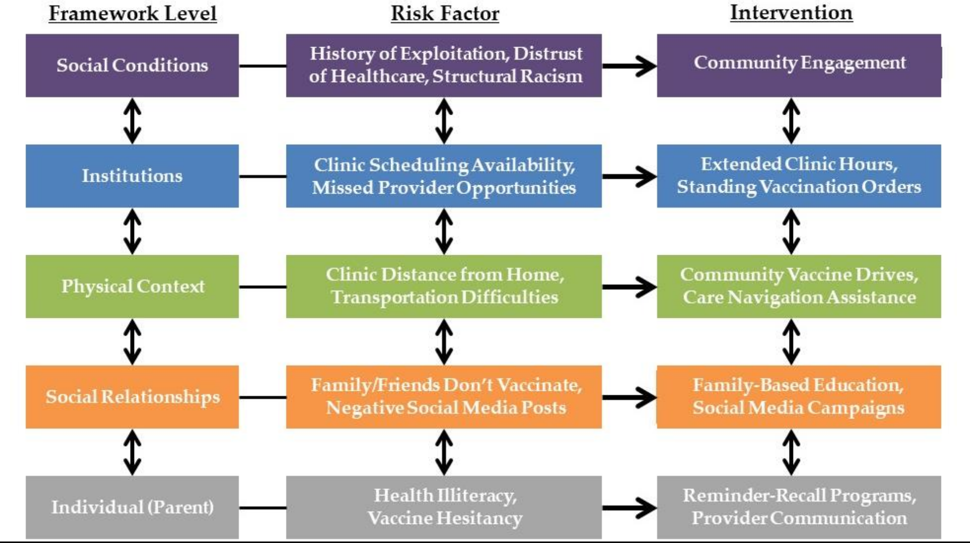

Previously, simply implementing interventions at individual or institutional risk levels reduced vaccination disparities. In 1995, Szilagyi used reminders to recall children in New York safety-net practices for overdue vaccines, noting a significant decrease in gaps between Black and White children after two years.[35] Zimmerman et al. developed a multi-component intervention in 2004 based on reminders, care navigators, and healthcare worker training. Implementing it in Pennsylvania safety-net clinics, he found significant increases in influenza vaccination rates, especially in clinics with the highest proportions of non-White children.[37,59] However, nearly 20 years later, a recent two-state pragmatic randomized trial found reminder-recall had low-level effects on increasing pediatric influenza vaccination.[39] Vaccine hesitancy has become a global threat,[18] and the COVID-19 pandemic has exacerbated distrust of vaccines in minority communities.[19] Indeed, in a 2019 national survey, Kempe et al. found that nearly 25% of parents are now hesitant about influenza vaccines.[38] **I believe reminders or standing orders are insufficient to overcome the rise of vaccine hesitancy and reduce inequity in vaccine-preventable diseases.**

Instead, community-based approaches that engage stakeholders to co-create culturally tailored interventions may be able to overcome vaccine hesitancy at individual, social, and structural risk levels. Prior work suggests Black adults desire tailored messaging about influenza vaccines that is grounded in Black culture, voice, and history.[40–42] *In my pilot engagement work, I found community members desired to hear personal stories about vaccines and vaccine-preventable diseases to increase their trust in vaccines, including the influenza vaccine specifically.*[9] **Digital stories are multi-modal narratives made by storytellers that synthesize images, video, voice, and text that create compelling accounts of experience and could improve confidence in influenza and other vaccines.**[43] **Digital Storytelling (DST) acts at multiple risk levels** (i.e. individual, social/interpersonal) to promote health practices[43] and is highly acceptable in minority cultures.[43,60,61] Furthermore, in recent randomized pragmatic trials of DST-based interventions, researchers have shown that digital stories have effectively improved and sustained blood pressure control in Black adults[44,45] and increased HPV vaccination

uptake in Korean college women.[46] **These preliminary studies give proof of concept to the stakeholder recommendation I uncovered.[9]**

III. Preliminary Studies/Progress Report:

See above.

IV. Research Methods

A. Outcome Measure(s):

Aim 1: Outcome measures will be qualitative, aiming at understanding stakeholders' perceptions of and preferences for digital stories and how they could be employed to reduce influenza vaccination disparities in Black children.

Aim 2: The outcome of aim 2 will be 10 professionally-made digital stories tailored to stakeholders' priorities, accompanying messaging from stakeholders and the CAAH, and a detailed implementation plan.

Aim 3: We will use a mixed-methods approach to evaluate primary outcomes of *acceptability*, *appropriateness*, *feasibility*, and *effectiveness*.[80,81] Caregiver independent variables will include age, sex (as a biological variable), education, income, race, ethnicity, insurance status, and scaled vaccine hesitancy score (range: 0-100, with 0 indicating not at all hesitant).[82] We will assess intervention caregivers' perceptions of digital storytelling (DST) *appropriateness* and *acceptability* with validated questions on a 5-point Likert scale, which read at a 5th grade level.[79] We will describe DST engagement using YouTube analytics, presenting the proportion of caregivers viewing digital stories (assessed via participant-specific URLs) and the median (range) of time videos were viewed. Child-level variables will include age, sex (as a biological variable), race, and ethnicity. To estimate pilot *effectiveness* for future R01 power calculations, our primary outcome will be child vaccination status at season's end (March 30th), defined in a binary manner (receipt of $\geq$ 1 dose by season's end versus unvaccinated). We chose this definition as our pilot data found parental vaccine hesitancy increased a child's risk of influenza non-vaccination but did not increase their risk of partial vaccination (vs. full).[7]

B. Description of Population to be Enrolled:

Aim 1: We will recruit up to 40 diverse stakeholders. To assist with recruitment, we have partnered with the Center for African American Health (CAAH) (see: Career Development Plan). We will recruit a sample of Black caregivers (n = 20; e.g., mothers, fathers, and grandparents) of children 6 months to 5 years old. We will recruit prospective participants through existing e-mail and phone contact lists at CAAH, using a combination of phone calls and emails (up to a total of three) and a standardized recruitment script. Through the same combination of phone calls and e-mails, we will leverage existing relationships to recruit clinicians, clinic managers, organizational vaccination champions, and ambulatory care services executives (n = 20) from within Denver Health and two primary care clinics (1 pediatric, 1 family medicine) located in

historically Black neighborhoods in Denver. If necessary, we will recruit additional caregivers or clinic and system stakeholders to reach thematic saturation. We will consent all participants and provide a $40 incentive.

Aim 2: We will recruit clinical and system stakeholders and Black caregivers during qualitative interviews (see: Aim 1) who are interested in co-creating and implementing a DST intervention in 2 safety-net clinics in historically Black neighborhoods. We will recruit approximately 10 participants: 6 Black caregivers of children 6 months to 5 years, 2 community leaders, and 2 clinic stakeholders.

Aim 3: We will pilot the intervention at 2 Denver Health primary care clinics in historically Black neighborhoods, aiming to recruit 200 predominantly Black caregivers. Caregivers will be eligible if their child is empaneled (i.e., had 1 well child visit in last 18 months), will be 6 months to 5 years old during the upcoming influenza season (10/1/24 to 3/30/25), and has no medical contraindications to vaccination. We will exclude caregivers whose first language is not English, as digital stories will be narrated in English, or are younger than 18 years. A research assistant will recruit Black caregiver-child dyads from 5/1/24 to 9/30/24 in clinic waiting rooms, or caregivers will be recruited with phone calls by Dr. Williams or a research assistant. Dr. Williams will screen individuals for eligibility by reviewing clinic schedules the day of recruitment for those individuals confirmed to have appointments the following day. Dr. Williams will call those individuals and offer them the ability to participate. See application for protocol review for full details. We will screen dyads for inclusion using EMR caregiver-reported race/ethnicity data, which is 95% complete at Denver Health (internal data) and confirmed each visit. Caregivers will complete a survey with demographic and vaccine hesitancy items and provide their cell phone number and e-mail address for intervention purposes. Caregivers will receive a $10 incentive for completion of an initial survey.

C. Study Design and Research Methods

Aim 1: This aim will be a qualitative analysis of semi-structured interviews with Black caregivers (n = 20) and clinic and organizational stakeholders (n = 20), informed by a multi-level disparities framework (Figure 2). We will use content analysis, a systematic yet flexible methodology, that offers rich insights for the purpose of intervention design.[67] Our group has recently used qualitative methods, including content analysis, to study diverse stakeholder attitudes toward practice-based and system-level interventions in other settings.[68,69]

We will create a semi-structured interview guide based on our adapted disparities research framework[48] (Figure 2) as it relates to best practices for health disparities intervention design.[70] At the start of each interview, we will show stakeholders a publicly-available digital story regarding Black health and solicit general feedback with open-ended questions. Then, we will probe stakeholders' perceptions of how digital stories might act across and within disparity risk levels (Figure 2) to complement or detract from other interventions to promote vaccination confidence. We will also explore priorities for story content, format, and length. Among Black caregivers, we will explore

perceptions of (i) the importance of digital stories about certain vaccines (i.e. stories specific to influenza vs. generic stories about vaccine-preventable diseases), (ii) inter-generational stories from older Black stakeholders, and (iii) stories from race-concordant and discordant healthcare providers. With organizational stakeholders, we will explore facilitators and barriers to the use of digital stories in clinical settings (i.e. cost, clinic flow, provider attitudes, etc.). Given the sensitive nature of attitudes toward vaccination, we will conduct individual interviews in lieu of focus groups. All interviews will be conducted via audio or video conferencing with a trained qualitative researcher (Dr. Williams or a race-concordant professional research assistant), last 45 minutes, and be audio-recorded and transcribed verbatim.

Aim 2: This aim will develop 10 digital stories accompanying messaging from stakeholders and the CAAH, and a detailed implementation plan. **Figure 3 details the BCT process, which will be co-facilitated by Dr. Williams and a Research Coordinator from the CAAH**. Dr. Williams will begin with an expert presentation on (i) pediatric influenza, (ii) knowledge, attitudes, and beliefs associated with disparities, (iii) DST and BCT, and (iv) qualitative study findings from Aim 1. The facilitator from the Center for African American Health will then help the group address 3 focusing questions: *(i) What is the preferred format for digital vaccine stories? (i.e. length, storyteller, focus, etc.), (ii) What messages about influenza and influenza vaccines should we highlight?, and (iii) How should we implement stories in 2 primary care practices to increase trust in vaccines?* This initial brainstorming session will be followed by a series of **6 half-day DST Workshop sessions spread over [3-6 months]** (Figure 4), facilitated by a race-concordant storyteller and Mr. Weinshenker (see: Expert Advisory Committee). Through this process, participants will learn the key elements of storytelling, think about their stories and reflect on peer feedback, receive expert audiovisual and editorial help, and share their final story with other BCT participants. This process will address the first two focusing questions. To address the final focusing question, participants will attend a

Fig. 3. Overview of BCT process.

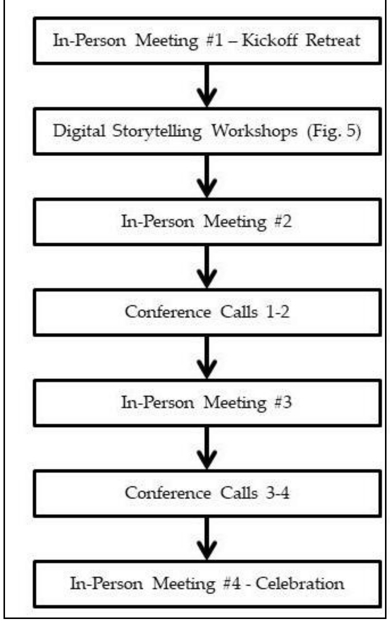

Figure 4. Overview of digital storytelling process, with descriptions of workshop phases.

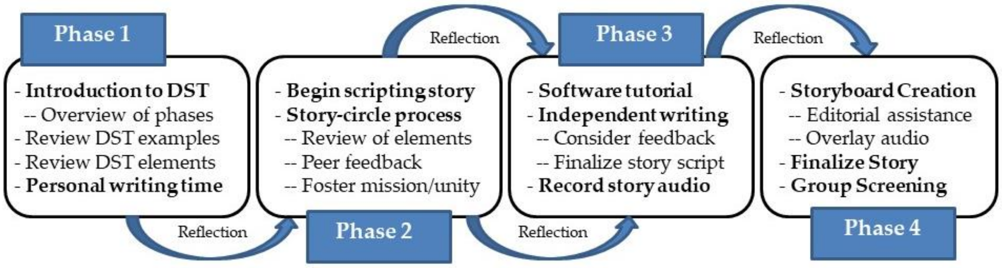

staggered series of quarterly in-person meetings and monthly conference calls (Figure 3). The goal of these sessions will be to refine any accompanying messaging and finalize an implementation plan for Aim 3 that aligns with stakeholders' priorities, leverages facilitators, and mitigates potential barriers.

Aim 3: **[***While this work is highly emergent in nature and exact implementation details are subject to stakeholder refinement, we anticipate the following structure.* On October 1st, dyads will be randomized to intervention or control groups (1:1 ratio). All caregivers will receive a series of 6 monthly texts and e-mails with health messaging and embedded videos. Control caregivers will receive messages encouraging reading with videos from Reach Out and Read Colorado. Intervention caregivers will receive messages with BCT influenza vaccine messaging, endorsed by the Center for African American Health, and Digital Stories. Intervention participants will view **6 different Digital Stories** delivered in the same number (n = 6) of texts and e-mails at the same frequency (monthly) as controls.**]** We will extract child vaccination status from Epic at season's end (March 30). Caregivers assigned to the intervention group (n = 100) will receive a follow-up survey with validated measures[79] assessing their perceptions of intervention *acceptability* and *appropriateness*. **[**Post-surveys will also ask caregivers to describe and rank the influence of other influenza-specific messaging (e.g., radio or TV ads, etc.) they viewed during the season. Messaging could moderate our intervention's treatment effect, although we expect intervention and control recipients to be similarly exposed. To complement caregiver perspectives, we will record Denver Health, county, and state influenza vaccine public health messaging efforts monthly. We will obtain message type, duration, and content directly from leaders of these entities with whom we work closely.**]** Lastly, we will hold qualitative focus groups on DST *feasibility* with 20 staff, offering $40 incentives.

D. Description, Risks and Justification of Procedures and Data Collection Tools:

D.1. Risks to Human Subjects

<u>Human Subjects Involvement, Characteristics, and Design:</u>

Aim 1: Overall, this study aim seeks to interview Black caregivers of young children and clinical, system, and administrative stakeholders to explore their perceptions and priorities for a digital storytelling intervention to reduce pediatric influenza vaccine disparities. We will enroll these individuals through snowball sampling using multiple contacts across e-mail, phone, and flyers. This has been feasible and effective in our work; we will recruit up to 40 individuals for 45-60 minute interviews.

Aim 2: We aim to recruit 6 Black caregivers, 2 community leaders and 2 clinic stakeholders will be recruited during qualitative interviews in Aim 2 to learn about DST and each create/record a story to share with communities.

Aim 3: We seek to recruit 200 caregivers (defined in Section B of this protocol) across 2 Denver Health primary care clinics and randomized into control or intervention arm. All caregivers will receive a series of texts, emails and surveys.

Study Procedures, Materials, and Potential Risks:

Aim 1: Semi-structured interviews of 45-60 minutes with caregivers and stakeholders as previously described. We will record all interviews and transcribe them, de-identifying them. There exists a risk of incidental re-identification based on specific topics of conversation. Study staff will review and code transcripts, and if incidental re-identification occurs, they will adjust transcripts as needed to preserve anonymity. Loss of confidentiality or privacy is very unlikely but a possible risk that will be mitigated by de-identifying transcripts and storing all files on a secure server accessible only via password, of which password strength will be sufficient. As the topic of interviews is somewhat controversial – vaccines – we will ensure all individuals are consented prior to the interview, provide adequate time or space during the interview for a participant to take a break (if needed), and remind interviewees at the end that consent is an ongoing process and that they can reach out to the study at any time to withdraw consent if they desire.

Aim 2: Bootcamp translation and digital storytelling workshops are involved processes that may be time-consuming. We will spread out the workshops in up to 6 half-day sessions and spread Bootcamp translation meetings over 3-6 months (as noted above) to allow for flexibility and less time commitment.

The final Pragmatic Trial will involve communication with caregivers and require them to complete surveys (as described in detail in section 3).

There is a potential risk of breach of personal information, that's why strict security measures will be followed (more in section D.2.).

D.2. Adequacy of Protection Against Risk

Informed Consent and Assent:

Aim 1: We will approach participants for our semi-structured interviews by trained study staff, in collaboration with trusted Care Navigators from the Center for African American Health, to obtain informed consent directly through conversation. We will outreach individuals by telephone, e-mail, and flyers. If on-line through a virtual meeting platform, we will ensure the meeting is password protected and that only the interviewer and interviewee have access to the meeting.

In addition to recruitment in collaboration with the Center for African American Health, trained study staff will recruit caregivers from 3 Denver Health clinics (Park Hill, Eastside, and Montbello). Dr. Williams will review pediatric clinic schedules one day in advance of planned recruitment activities. Dr. Williams will call parents of children who have a scheduled appointment whose age is 6 months to 5 years and whose electronic health records indicate that their race is Black or African American. Parents will be informed of the opportunity to participate in this Digital Storytelling Project using the telephone script as above and offered an opportunity to participate before or after their appointment time. Caregivers will be informed that their participation has no impact on whether or not they will receive care the following day and is completely voluntary. This recruitment process will happen at outpatient Denver Health clinics at which the principal investigator does not work (PI works at Webb Pediatrics).

Finally, we will post our already-approved flyer (version 2.24.23) in community centers, recreation centers, youth centers, and libraries in the immediate neighborhoods surrounding the aforementioned clinics. Flyers contain all information pertinent to the study and e-mail contact information for the principal investigator.

Aim 2: A postcard consent will be used for BCT participants attending workshop sessions. Anyone creating a digital story that will be shared will sign a consent before participating.

Aim 3: Caregivers participating in the Pragmatic Trial will be recruited in a clinic waiting room by a research assistant who will explain the study and ask if the caregiver is interested in participating, or contacted by the research assistant or Dr. Williams the day prior to potential recruitment. If interested, caregivers will complete an (entry) survey with demographic and vaccine hesitancy items and provide their cell phone number and e-mail address for intervention purposes. The entry survey will include postcard consent text. The participants will be receiving texts, emails and completing surveys only. If there is not enough time in the waiting room for recruitment (based on experience after the first few weeks), research staff will call 1 day ahead of the appointment to offer participation in the study. The consent process (and later the entry survey) will be completed in a private setting at the clinic or over the phone with research staff.

We will obtain a waiver of documentation of consent for all interviews and caregivers receiving texts, emails and surveys. Participants will be given a copy of the e-mail or telephone consent script that includes all contact information for the principal investigator and IRB of record. Caregivers will verbally agree to participate after being read the postcard consent. Study staff will answer all questions before any data are collected, including the referring of any incompletely understood or partially answered questions to Dr. Williams. The strictly voluntary nature of the study will be clearly stated in any recruitment materials (e.g. flyers) and during the consent process. We will deidentify all interviews immediately once transcribed. All surveys will be deidentified (each survey will include an ID number that will be linked to a participant only at Denver Health server accessible by research staff). Collected phone numbers and email addresses on a paper entry survey will be stored in a locked location at Denver Health. Electronic data will be stored only on Denver Health secured drives. Demographic details for participants will be collected, but specific identifiers such as birthdates or names will not be part of the analytic dataset. Participants will be informed that under no circumstances will identifiable data be available to anyone outside of the study team.

<u>Protections Against Risk:</u> Several measures will be taken to ensure confidentiality of the information collected. Semi-structured interviews will be de-identified during transcription. All transcripts will be stored on a secure, password-protected folder on encrypted serves at Denver Health and ACCORDS, and only appropriate members of the study team will have direct access to these files. Prior to seeking participants for any aspect of the study, Human Subjects Research approval will be obtained from the IRB at

the University of Colorado (COMIRB). All study procedures will comply with HIPPAA, federal, and institutional regulations governing human subjects research. For all research, the following additional protections will be implemented:

- All investigators involved in the study aim will have completed either the Program for Education and Evaluation in Responsible Research and Scholarship (PEERRS) or the Collaborative Institutional Training Initiative (CITI) online training courses related to human subject protections. These courses fulfill all NIH requirements for human subjects research training.
- Study activities will not commence until approval has been granted from COMIRB.
- We will obtain annual re-approvals of our research for continuation of this study.
- We will report any adverse events that occur immediately to COMIRB in accordance with federal and institutional policies

Vulnerable subjects: Overall, this study presents minimal risk for participants, and all efforts will be made to protect and minimize risks to vulnerable subjects throughout this study. *This study will not involve fetuses, prisoners, children or institutionalized individuals*. We will recruit *women* and *minorities* in this Study. We do not expect any increased risk for women and minorities who participate in this study, but all efforts will be made to minimize risk through the methods described above for all participants. No exposures to medications, treatments, or devices will occur.

D.3. Potential Benefits of the Proposed Research to Research Participants and Others

There is limited immediate benefits to participants in this study. However, the results of the first Study Aim will inform the development of digital stories and associated messaging in Aim 2 (which will later be leveraged in an Aim 3 pilot pragmatic trial). Thus, we expect the benefits of interviewees' insights to accrue in subsequent years, especially as it may decrease the burden of influenza morbidity and mortality in Denver and beyond. This will benefit caregivers of young children, the physicians who care for them, and the administrative and system stakeholders who support infrastructure needed for clinical care at Denver Health.

E. Potential Scientific Problems:

(1) Limited recruitment. With content analysis, best practice suggests 30-40 interviews are needed for thematic saturation.[67] Our community partners have committed to assisting with recruitment to reach saturation. We will also pursue recruit in office settings to complement efforts from our community partners and ensure we get a robust sample. With regard to aim 3, we collected pilot data in the same clinic and 2 others from 255 caregivers of 2 year-olds in 6 months.[6] By extending inclusion criteria to ages 6 months to 5 years, we are confident we can recruit 200 dyads. *(2) Lack of transferability*. Caregivers' perspectives may not be transferable to other Black communities outside Colorado, especially those who also identify as Latino.[71] Yet, this study is an important first step in understanding how DST complements existing interventions to reduce vaccination disparities. *(3) Poor perceived acceptability, appropriateness, or feasibility*. If our analyses

suggest we need to improve intervention feasibility, acceptability, or appropriateness, we will work with community and clinical stakeholders to iteratively modify and pilot-test the adapted intervention.

F. Data Analysis Plan:

A team of at least two qualitatively trained investigators, one of whom identifies as Black or African American, will inductively code transcripts in an iterative fashion, resolving discrepancies through discussion and consensus. Investigators will first independently review a set of transcripts (n = 5) to inductively develop the initial codebook. Then, collaboratively, the investigators will consolidate and reconcile the codebook and independently apply it to another set of transcripts (n = 5). This process will continue until a final codebook is agreed upon, at which time the codebook will be applied to the remainder of the interviews. At least three investigators will review coded transcripts and examine codes within and across categories to create themes. All coded data will be entered into ATLAS.ti software, version 8.4 (Berlin, Germany) for data management. To ensure trustworthiness of our findings, we will (1) engage a multidisciplinary team throughout the project, (2) maintain an audit trail, and (3) share our final themes and interpretations of them with three individuals from different stakeholder levels, incorporating their feedback into our final analyses.

For quantitative analyses, we will calculate descriptive statistics of caregivers' demographics and vaccine hesitancy scores. We will then describe intervention caregivers' perceptions of *acceptability* and *appropriateness;* as set cut points for *acceptability and appropriateness* do not yet exist, we plan to use ≥ 70% agreement as with prior work by our group. Using these cut points, we will fit univariable and multivariable regression models to estimate the association between covariates (e.g., demographics, vaccine hesitancy score) and whether a caregiver perceived the intervention as *acceptable* or *appropriate*.

For our quantitative outcome of pilot *effectiveness*, we will use an intention-to-treat approach comparing the proportion of children of caregivers randomized to digital stories versus literacy videos who received ≥1 vaccine dose by season's end. We will fit univariable and multivariable regression models to explore associations with caregiver demographics and parental vaccine hesitancy scores. To address instances of video non-viewing, we will repeat these analyses between caregivers whose unique Digital Story links were accessed – i.e., presumably viewed – and caregivers in the control group. Analyses will be performed in R.

We performed sample size calculations based on the precision with which we could estimate the treatment effect, as measured by the difference in proportion vaccinating between intervention and control arms. We hypothesize our estimated pilot effect size will be ≥ 0.10, or modest for a vaccination trial. With n = 200, we expect to attain a margin of error (equal to one half of the width of a 95% confidence interval) for the estimated treatment effect of approximately 0.14.

G. Summarize Knowledge to be Gained:

Results from this study will significantly advance our understanding of perceptions of and priorities for Digital Storytelling interventions to reduce vaccination disparities in Black children. This will enhance the development of Digital Stories in later study aims and also provide a template for other researchers who might seek to use Digital Stories to increase vaccination confidence with other minority groups or different vaccines. Furthermore, should interviews suggest Digital Storytelling is highly acceptable to providers and caregivers alike, it could open the door for additional interventions grounded in stories for other conditions in adults and children. Thus, as the study presents minimal risks to participants, the importance of the knowledge to be gained is valuable enough that the risks are reasonable to assume in relation.

H. References:

1. Epperson S, Blanton L, Kniss K, et al. Influenza Activity - United States, 2013-14 Season and Composition of the 2014-15 Influenza Vaccines. *Morb Mortal Wkly Rep*. 2014;63(22):483-490. doi:10.1016/j.annemergmed.2014.08.002
2. Williams JTB, Cunningham MA, Wilson KM, Rao S. Rising Oseltamivir Use Among Hospitalized Children in a Postpandemic Era. *Hosp Pediatr*. 2016;6(3):172-178. doi:10.1542/hpeds.2015-0126
3. Rao S, Williams JTB, Torok MR, Cunningham MA, Glodè MP, Wilson KM. Missed Opportunities for Influenza Vaccination Among Hospitalized Children With Influenza at a Tertiary Care Facility. *Hosp Pediatr*. 2016;6(9):513-519. doi:10.1542/hpeds.2015-0112
4. Rao S, Torok MR, Bagdure D, et al. A Comparison of H1N1 Influenza Among Pediatric Inpatients in the Pandemic and Post Pandemic Era. *J Clin Virol*. 2015;Oct(71):44-50.
5. Williams JTB, O'Leary ST. Denver Religious Leaders' Vaccine Attitudes, Practices, and Congregational Experiences. *J Relig Heal*. 2019;58(4):1356-1367.
6. Williams JTB, Rice JD, Lou Y, et al. Parental Vaccine Hesitancy and Vaccination Disparities in a Safety-Net System. *Pediatrics*. 2021;147(2):e2020010710.
7. Williams JTB, Fisher MP, Bayliss EA, et al. Clergy attitudes toward vaccines and vaccine advocacy : a qualitative study. *Hum Vaccin Immunother*. 2020;16(11):2800-2808. doi:10.1080/21645515.2020.1736451
8. Williams JTB, Rice J, Cox-Martin M, Bayliss EA, O'Leary ST. Religious vaccine exemptions in kindergartners: 2011-2018. *Pediatrics*. 2019;144(6):e20192710. doi:10.1542/peds.2019-2710
9. Williams JTB, Miller A, O'Leary ST. Sacred or secular? Exploring religious Coloradans' questions about vaccines. *Vaccine*. 2020;38(45):6971-6974. doi:10.1016/j.vaccine.2020.09.034
10. Measles Cases and Outbreaks. Centers for Disease Control and Prevention. Published 2020. https://www.cdc.gov/measles/cases-outbreaks.html
11. Williams JTB, Nussbaum AM. Minister to their Instruction: Revisiting the Minister-Vaccinator Rowland Hill. *Pediatrics*. 2018;142(6):e20181021.
12. Williams JTB, Nussbaum AM, O'Leary ST. Building trust: Clergy and the call to

eliminate religious exemptions. *Pediatrics*. 2019;144(4):e20190933. doi:10.1542/peds.2019-0933

13. Williams JTB. Measles, Mumps, And Communion: A Vision For Vaccine Policy. *Health Aff (Millwood)*. 2019;38(11):1944-1947. doi:10.1377/hlthaff.2019.00446
14. Williams JTB, Nussbaum AM. Reverend Rowland Hill and a Role for Religious Leaders in Vaccine Promotion. *Am J Public Health*. 2019;109(5):697-698. doi:10.2105/AJPH.2019.1095695
15. Williams JTB. The School Vaccination Assessment Program: A Physician's Perspective. *Am J Public Health*. 2020;110(7):927-928.
16. Williams JTB, Nussbaum AM, O'Leary ST. What about Clergy? A Surprisingly Absent "Cue to Action" in McCoy et al. (2018). *Vaccine*. 2019;37(38):5665-5666.
17. Williams JTB, O'Leary ST, Nussbaum AM. Caring for the Vaccine-Hesitant Family: Evidence-Based Alternatives to Dismissal. *J Pediatr*. 2020;224(September):137-140. doi:10.1016/j.jpeds.2020.05.029
18. Ten Threats to Global Health in 2019. World Health Organization. Published 2019. Accessed February 20, 2020. https://www.who.int/news-room/feature-stories/ten-threats-to-global-health-in-2019
19. Webb Hooper M, Napoles AM, Perez-Stable EJ. COVID-19 and Racial/Ethnic Disparities. *JAMA*. 2020;323(24):2466-2467. doi:10.1002/jclp.20757
20. Grohskopf LA, Liburd LC, Redfield RR. Addressing Influenza Vaccination Disparities During the COVID-19 Pandemic. *J Am Med Assoc*. 2020;324(11):1029-1030.
21. Crocker-Buque T, Edelstein M, Mounier-Jack S. Interventions to reduce inequalities in vaccine uptake in children and adolescents aged < 19 years: A systematic review. *J Epidemiol Community Health*. 2016;71(1):87-97. doi:10.1136/jech-2016-207572
22. Disease Burden of Influenza. Centers for Disease Control and Prevention. Published 2020. Accessed January 2, 2021. https://www.cdc.gov/flu/about/burden/index.html
23. Dee DL, Bensyl DM, Gindler J, et al. Racial and Ethnic Disparities in Hospitalizations and Deaths Associated with 2009 Pandemic Influenza A (H1N1) Virus Infections in the United States. *Ann Epidemiol*. 2011;21(8):623-630. doi:10.1016/j.annepidem.2011.03.002
24. Navaranjan D, Rosella LC, Kwong JC, Campitelli M, Crowcroft N. Ethnic disparities in acquiring 2009 pandemic H1N1 influenza: A case-control study. *BMC Public Health*. 2014;14(1):1-10. doi:10.1186/1471-2458-14-214
25. Yousey-Hindes KM, Hadler JL. Neighborhood socioeconomic status and influenza hospitalizations among children: New Haven County, Connecticut, 2003-2010. *Am J Public Health*. 2011;101(9):1785-1789. doi:10.2105/AJPH.2011.300224
26. Kenneth D. Kochanek, M.A., Sherry L. Murphy, B.S., Jiaquan Xu, M.D., and Elizabeth Arias PD. *National Vital Statistics Reports: Deaths 2017*. Vol 68.; 2017. doi:10.1111/mec.13536.Application
27. Iwane MK, Chaves SS, Szilagyi PG, et al. Disparities between black and white children in hospitalizations associated with acute respiratory illness and laboratory-confirmed influenza and respiratory syncytial virus in 3 US counties -

2002-2009. *Am J Epidemiol*. 2013;177(7):656-665. doi:10.1093/aje/kws299

28. Maldonado YA, Zaoutis TE, Banerjee R, et al. Recommendations for prevention and control of influenza in children, 2019–2020. *Pediatrics*. 2019;144(4):2019-2020. doi:10.1542/peds.2019-2478
29. Zhai Y, Santibanez TA, Kahn KE, Srivastav A. Parental-Reported Full Influenza Vaccination Coverage of Children in the U.S. *Am J Prev Med*. 2017;52(4):e103-e113. doi:10.1016/j.amepre.2016.10.040
30. Yoo BK, Berry A, Kasajima M, Szilagyi PG. Association between medicaid reimbursement and child influenza vaccination rates. *Pediatrics*. 2010;126(5). doi:10.1542/peds.2009-3514
31. Hill HA, Singleton JA, Yankey D, Elam-Evans LD, Pingali SC, Kang Y. Vaccination Coverage by Age 24 Months Among Children Born in 2015 and 2016 - National Immunization Survey-Child, United States, 2016-2018. *MMWR Morb Mortal Wkly Rep*. 2019;68(41):913-918. doi:10.15585/mmwr.mm6841e2
32. Flannery B, Reynolds SB, Blanton L, et al. Influenza vaccine effectiveness against pediatric deaths: 2010-2014. *Pediatrics*. 2017;139(5):e20164244. doi:10.1542/peds.2016-4244
33. Flu Vaccination Coverage, United States, 2018-2019 Influenza Season. Centers for Disease Control and Prevention2. Published 2019. Accessed February 20, 2020. https://www.cdc.gov/flu/fluvaxview/coverage-1819estimates.htm
34. Hofstetter AM, Natarajan K, Rabinowitz D, et al. Timeliness of pediatric influenza vaccination compared with seasonal influenza activity in an urban community, 2004-2008. *Am J Public Health*. 2013;103(7):e50-58. doi:10.2105/AJPH.2013.301351
35. Szilagyi PG, Schaffer S, Shone L, et al. Reducing geographic, racial, and ethnic disparities in childhood immunization rates by using reminder/recall interventions in urban primary care practices. *Pediatrics*. 2002;110(5):e58. doi:10.1542/peds.110.5.e58
36. Zimmerman RK, Nowalk MP, Lin CJ, et al. Cluster randomized trial of a toolkit and early vaccine delivery to improve childhood influenza vaccination rates in primary care. *Vaccine*. 2014;32(29):3656-3663. doi:10.1016/j.vaccine.2014.04.057
37. Nowalk MP, Lin CJ, Hannibal K, et al. Increasing childhood influenza vaccination: A cluster randomized trial. *Am J Prev Med*. 2014;47(4):435-443. doi:10.1016/j.amepre.2014.07.003
38. Kempe A, Saville AW, Albertin C, et al. Parental hesitancy about routine childhood and influenza vaccinations: A national survey. *Pediatrics*. 2020;146(1):e20193852. doi:10.1542/peds.2019-3852
39. Kempe A, Saville AW, Albertin C, Helmkamp L, Zhou X, Vangela S DL, Tseng CH, Campbell JD, Whittington M, Gurfinkel D, Roth H, Hoefer D SP. Centralized Reminder/Recall to Increase Influenza Vaccination Rates: A Two-State Pragmatic Randomized Trial. *Acad Pediatr*. 2020;20(3):374-383.
40. Freimuth VS, Jamison AM, An J, Hancock GR, Quinn SC. Determinants of Trust in the Flu Vaccine for African Americans and Whites. *Soc Sci Med*. 2017;193:70-79.
41. Jamison AM, Quinn SC, Freimuth VS. "You don't trust a government vaccine": Narratives of institutional trust and influenza vaccination among African American and white adults. *Soc Sci Med*. 2019;221:87-94.

doi:10.1016/j.socscimed.2018.12.020
42. Quinn SC. African American adults and seasonal influenza vaccination: Changing our approach can move the needle. *Hum Vaccines Immunother*. 2018;14(3):719-723. doi:10.1080/21645515.2017.1376152
43. Gubrium A. Digital Storytelling: An Emergent Method for Health Promotion Research and Practice. *Health Promot Pract*. 2009;10(2):186-191. doi:10.1177/1524839909332600
44. Houston TK, Allison JJ, Sussman M, et al. Culturally appropriate storytelling to improve blood pressure: a randomized trial. *Ann Intern Med*. 2011;154(2):77-84.
45. Myers KR, Green MJ. Storytelling: a novel intervention for hypertension. *Ann Intern Med*. 2011;154(2):129-130.
46. Kim M, Lee H, Kiang P, et al. A Storytelling Intervention in a Mobile, Web-Based Platform: A Pilot RCT to Evaluate the Preliminary Effectiveness to Promote HPV Vaccination in Korean American College Women. *Heal Educ Behav*. 2020;47(2):258-263. doi:10.1177/1090198119894589.A
47. Wallerstein N, Duran B. Community-based participatory research contributions to intervention research: The intersection of science and practice to improve health equity. *Am J Public Health*. 2010;100(SUPPL. 1):40-46. doi:10.2105/AJPH.2009.184036
48. Alvidrez J, Castille D, Laude-Sharp M, Rosario A, Tabor D. The National Institute on Minority Health and Health Disparities Research Framework. *Am J Public Health*. 2019;109(S1):S16-S20. doi:10.2105/AJPH.2018.304883
49. Norman N, Bennett C, Cowart S, et al. Boot Camp translation: A method for building a community of solution. *J Am Board Fam Med*. 2013;26(3):254-263. doi:10.3122/jabfm.2013.03.120253
50. Westfall JM, Zittleman L, Felzien M, et al. Reinventing the wheel of medical evidence: How the boot camp translation process is making gains. *Health Aff*. 2016;35(4):613-618. doi:10.1377/hlthaff.2015.1648
51. Brenner RA, Simons-Morton BG, Bhaskar B, Das A, Clemens JD. Prevalence and predictors of immunization among inner-city infants: A birth cohort study. *Pediatrics*. 2001;108(3):661-670. doi:10.1542/peds.108.3.661
52. Wood D, Donald-Sherbourne C, Halfon N, et al. Factors related to immunization status among inner-city Latino and African-American preschoolers. *Pediatrics*. 1995;96(2):295-301.
53. Bobo JK, Gale JL, Thapa PB, Wassilak SGF. Risk factors for delayed immunization in a random sample of 1163 children from Oregon and Washington. *Pediatrics*. 1993;91(2):308-314.
54. Miller LA, Hoffman RE, Baron AE, Marine WM, Melinkovich P. Risk factors for delayed immunization against measles, mumps, and rubella in Colorado two-year-olds. *Pediatrics*. 1994;94(2 I):213-219.
55. Saville AW, Szilagyi P, Helmkamp L, Albertin C, Gurfinkel D VS, Dickinson LM, Zhou X, Roth H KA. Potential Strategies to Achieve Universal Influenza Vaccination for Children: Provider Attitudes in Two States. *Acad Pediatr*. 2018;18(8):873-881.
56. Warnecke RB, Oh A, Breen N, et al. Approaching health disparities from a population perspective: The National Institutes of Health Centers for Population

Health and Health Disparities. *Am J Public Health*. 2008;98(9):1608-1615. doi:10.2105/AJPH.2006.102525

57. Johnson TJ. Intersection of bias, structural racism, and social determinants with health care inequities. *Pediatrics*. 2020;146(2):e2020003657. doi:10.1542/peds.2020-003657

58. Krieger N. ENOUGH: COVID-19, Structural Racism, Police Brutality, Plutocracy, Climate Change-and Time for Health Justice, Democratic Governance, and an Equitable, Sustainable Future. *Am J Public Health*. 2020;110(11):1620-1623. doi:10.2105/AJPH.2020.305886

59. Zimmerman RK, Nowalk MP, Lin CJ, et al. Interventions over 2 years to increase influenza vaccination of children aged 6-23 months in inner-city family health centers. *Vaccine*. 2006;24(10):1523-1529. doi:10.1016/j.vaccine.2005.10.016

60. Larkey LK, Hecht M. A model of effects of narrative as culture-centric health promotion. *J Health Commun*. 2010;15(2):114-135. doi:10.1080/10810730903528017

61. Murphy S, Frank LB, Chatterjee J, Baezconde-Garbanati L. [Narrative] Transportation and Emotion in Reducing Health Disparities. *J Commun*. 2013;63(1):1-19. doi:10.1111/jcom.12007.Narrative

62. Graham LF, Scott L, Lopeyok E, Douglas H, Gubrium A, Buchanan D. Outreach Strategies to Recruit Low-Income African American Men to Participate in Health Promotion Programs and Research: Lessons From the Men of Color Health Awareness (MOCHA) Project. *Am J Mens Health*. 2018;12(5):1307-1316. doi:10.1177/1557988318768602

63. Gubrium AC, Lowe S, Douglas H, Scott L, Buchanan D. Participant Engagement and Ethical Digital Storytelling: The MOCHA Moving Forward Study. *Int Q Community Health Educ*. 2020;40(4):263-271. doi:10.1177/0272684X19862931

64. Lott BE, Okusanya BO, Anderson EJ, et al. Interventions to increase uptake of Human Papillomavirus (HPV) vaccination in minority populations: A systematic review. *Prev Med Reports*. 2020;19(July):101163. doi:10.1016/j.pmedr.2020.101163

65. Universal Influenza Vaccine Research. National Institute of Allergy and Infectious Diseases. Published 2020. Accessed January 1, 2021. https://www.niaid.nih.gov/diseases-conditions/universal-influenza-vaccine-research

66. Moullin JC, Dickson KS, Stadnick NA, Rabin B, Aarons GA. Systematic review of the Exploration, Preparation, Implementation, Sustainment (EPIS) framework. *Implement Sci*. 2019;14(1):1-16. doi:10.1186/s13012-018-0842-6

67. Hsieh HF, Shannon SE. Three approaches to qualitative content analysis. *Qual Health Res*. 2005;15(9):1277-1288. doi:10.1177/1049732305276687

68. Fisher MP, Gurfinkel D, Szilagyi PG, et al. Supporting and sustaining centralized reminder/recall for immunizations: Qualitative insights from stakeholders. *Vaccine*. 2019;37(44):6601-6608. doi:10.1016/j.vaccine.2019.09.055

69. Reno JE, O'Leary ST, Pyrzanowski J, Lockhart S, Thomas J, Dempsey AF. Evaluation of the Implementation of a Multicomponent Intervention to Improve Health Care Provider Communication About Human Papillomavirus Vaccination. *Acad Pediatr*. 2018;18(8):882-888. doi:10.1016/j.acap.2018.08.004

70. Agurs-Collins T, Persky S, Paskett ED, et al. Designing and Assessing Multilevel

Interventions to Improve Minority Health and Reduce Health Disparities. *Am J Public Health*. 2019;109(S1):S86-S93. doi:10.2105/AJPH.2018.304730

71. Strauss A, Corbin J. *Basics of Qualitative Research: Techniques and Procedures for Developing Grounded Theory*. 3rd ed. Sage; 2008.

72. English AF, Dickinson LM, Msph LZ, et al. A community engagement method to design patient engagement materials for cardiovascular health. *Ann Fam Med*. 2018;16:S38-S64. doi:10.1370/afm.2173

73. Zittleman L, Emsermann C, Dickinson M, et al. Increasing colon cancer testing in rural Colorado: Evaluation of the exposure to a community-based awareness campaign. *BMC Public Health*. 2009;9:1-9. doi:10.1186/1471-2458-9-288

74. John MW, Zittleman L, Sutter C, et al. Testing to prevent colon cancer: Results from a rural community intervention. *Ann Fam Med*. 2013;11(6):500-507. doi:10.1370/afm.1582

75. Bender BG, Dickinson P, Rankin A, Wamboldt FS, Zittleman L, Westfall JM. The Colorado asthma toolkit program: A practice coaching intervention from the high plains research network. *J Am Board Fam Med*. 2011;24(3):240-248. doi:10.3122/jabfm.2011.03.100171

76. Brewer SE, Nederveld A, Simpson M. Engaging communities in preventing human papillomavirus-related cancers: Two boot camp translations, Colorado, 2017-2018. *Prev Chronic Dis*. 2020;17(E02):1-6. doi:10.5888/pcd17.190250

77. Opel DJ, Taylor JA, Mangione-Smith R, et al. Development of a survey to identify vaccine-hesitant parents. *Hum Vaccin*. 2011;7(4):419-425. doi:10.1016/j.vaccine.2011.06.115

78. Weiner BJ, Lewis CC, Stanick C, et al. Psychometric assessment of three newly developed implementation outcome measures. *Implement Sci*. 2017;12(1):1-12. doi:10.1186/s13012-017-0635-3

79. Glasgow RE. What Does It Mean to Be Pragmatic? Pragmatic Methods, Measures, and Models to Facilitate Research Translation. *Heal Educ Behav*. 2013;40(3):257-265. doi:10.1177/1090198113486805

80. Ford I, Norrie J. Pragmatic trials. *N Engl J Med*. 2016;375(5):454-463. doi:10.1056/NEJMra1510059

81. Opel DJ, Taylor JA, Mangione-Smith R, et al. Validity and reliability of a survey to identify vaccine-hesitant parents. *Vaccine*. 2011;29(38):6598-6605. doi:10.1016/j.vaccine.2011.06.115

82. Healthy People 2030: Increase the Proportion of People who get the Flu Vaccine Every Year (IID-09). Office of Disease Prevention and Health Promotion. Published 2020. https://health.gov/healthypeople/objectives-and-data/browse-objectives/vaccination/increase-proportion-people-who-get-flu-vaccine-every-year-iid-09

83. Eunice Kennedy Shriver National Institute of Child Health and Human Development (NICHD): Mission. NIH. Published 2020. Accessed January 2, 2021. https://www.nih.gov/about-nih/what-we-do/nih-almanac/eunice-kennedy-shriver-national-institute-child-health-human-development-nichd#:~:text=The mission of the Eunice,potential to live healthy and
