# Supplementary material for: Narrative Reminder Recall to Improve Pediatric Influenza Vaccination: A Pilot Randomized Clinical Trial
Source: JAMA Netw Open. 2026 Jan 6;9(1):e2552149. doi: 10.1001/jamanetworkopen.2025.52149 (PMC12776203; doi:10.1001/jamanetworkopen.2025.52149)
Supplement: Supplement 2. — Data Sharing Statement [file jamanetwopen-e2552149-s002.pdf]

# Data Sharing Statement

Williams. Narrative Reminder Recall to Improve Pediatric Influenza Vaccination. *JAMA Netw Open*. Published January 06, 2026. doi:10.1001/jamanetworkopen.2025.52149

**Data**
**Additional Information:** ClinicalTrials.gov; NCT06274359.
https://clinicaltrials.gov/study/NCT06274359?intr=digital%20storytelling&rank=2
**Data available:** Yes
**Data types:** Deidentified participant data
**How to access data:** Deidentified data will be made available upon reasonable request to the PI.
**When available:** With publication

**Supporting Documents**
**Document types:** None

**Additional Information**
**Who can access the data:** To researchers whose proposed use of the data has been approved.
**Types of analyses:** For research purposes approved by an institutional review board.
**Mechanisms of data availability:** With investigator support, after approval of a proposal.
**Any additional restrictions:** n/a
